# Editorial: New insights in cellular and molecular biology of cancer stem cells

**DOI:** 10.3389/fgene.2023.1297971

**Published:** 2023-10-02

**Authors:** Karla Vazquez-Santillan, Ximena Bustamante-Marin, Elizabeth Ortiz-Sanchez

**Affiliations:** ^1^ Laboratorio de Innovación en Medicina de Presición, Instituto Nacional de Medicina Genómica, Secretaría de Salud, Mexico City, Mexico; ^2^ Department of Nutrition, University of North Carolina, Chapel Hill, NC, United States; ^3^ Nutrition Research Institute, University of North Carolina, Chapel Hill, NC, United States; ^4^ Subdirección de Investigación Básica, Instituto Nacional de Cancerología, Secretaría de Salud, Mexico City, México

**Keywords:** cancer, stem cells, self-renewal, signaling pathways, tumor microenvironment

Cancer stem cells residing in tumor mass exhibit two main properties: self-renewal and differentiation. Self-renewal is the process by which a stem cell divides to preserve its stemness traits in at least one daughter cell per division. A growing number of studies show that CSCs accomplish self-renewal by both symmetric and asymmetric cell division. Symmetric cell division produces two cancer stem cells with self-renewal abilities, while asymmetric cell division produces 2 cells with different fates (an undifferentiated cancer stem cell and a cell committed to differentiate). Differentiation is a process by which CSCs give rise to more differentiated cells incapable of sustaining tumor growth. Differentiation endows CSCs with the capacity to provide a continuous supply of heterogeneous tumor cells to maintain uninterrupted tumor growth (Batlle and Clevers, 2017).

The identification and characterization of cancer stem cells have been a key step in cancer research. Substantial efforts are dedicated to unraveling the intricate biology of this cell fraction. CSCs possess a distinct set of functional and biological attributes that distinguish them from other non-stem cells within tumors. These cells are identified by the presence or absence of various stem cell markers that are heterogeneously distributed across different tumors. These markers include cell surface proteins (CD44, CD24, ESA, CD133), cytoplasmic enzymes (ALDH1A3, ALDH8A1, ALDH1A1), and nuclear transcription factors (SOX2, OCT4, NANOG, KLF4) (Batlle and Clevers, 2017).

CSCs of solid tumors exhibit a plethora of distinctive features including the ability to survive and form spheres under low adherent conditions, the potential to initiate tumors in immunodeficient animal models, even when inoculated in minimal quantities, and the capacity to efflux drugs owing to the overexpression of ABC drug transporters. These characteristics have been extensively studied in many types of tumors; however, little has been explored about the inner membrane composition of CSCs and the implication of these features on the functionality and behavior of CSCs. A study conducted by Ritter et al. show that stem cells possess the innate capacity to internalize a fluorescent double-stranded DNA through electrostatic interactions facilitated by the attraction between the negatively charged dsDNA and the positively charged stem cells. In addition, the authors indicate that certain surface proteins present in the CSCs play a pivotal role in facilitating the binding of the fluorescent DNA. These molecules possess a heparin-binding domain characterized by a cluster of Lysine/Arginine residues, thereby forming a positively charged groove. Furthermore, the internalization of the dsDNA was shown to occur through a mechanism involving caveolae and/or clathrin-dependent endocytosis and micropinocytosis. This manuscript provides evidence for the existence of a well-developed glycocalyx on CSCs, featuring a diverse array of membrane-anchored proteins associated with lipid rafts and clathrin complexes with the ability to bind DNA (Ritter et al.).

Similarly, another crucial attribute that influences the fate of cancer stem cells is the tumor microenvironment (TMA). In an interesting study by Bustamante-Marin and Capel, it was shown a relationship between the genetics of male stem germ cells and their response to the microenvironment. Heterozygosity of the potent modifier of tumor incidence *Ter*, a point mutation in the dead-end homolog 1 gene (*Dnd1* ^*Ter/+*^) renders male germ cells susceptible to tumorigenic transformation. In 129/SvJ *Dnd1* ^*Ter/+*^ mice, ∼80% of the unilateral teratomas arise in the left testis. Interestingly, as previously shown, the vascular architecture differences between the right and left adult testes are differentially oxygenated, and the left testes express higher levels of HIF-1α (Bustamante-Marin and Capel). Here, the authors showed that systemic exposure to hypoxia during a specific window of embryonic development of male stem germ cells 129/SvJ *Dnd1* ^*Ter/+*^ (E13.8–E14.3) eliminated the left bias of teratoma development and increased their susceptibility to develop bilateral teratoma from 3% to 64%, evaluated as E-Cadherin clusters in both gonads. The phenotype correlated with high expression of pluripotency genes *Oct4*, *Sox2,* and *Nanog*, elevated activity of the Nodal signaling pathway, and suppression of germ cell mitotic arrest (Bustamante-Marin and Capel). The understanding of the influence of the testicular microenvironment on the fate of male germ cells could lead to the identification of therapeutic targets to develop successful preventions and treatments for testicular cancer.

CSCs are clinically relevant since accumulating evidence has shown that these cell subpopulations provoke tumor progression, treatment resistance, metastasis, and recurrence. In light of the resistance that has emerged in patients towards all anti-neoplastic therapies, it becomes necessary to ascertain the “Achilles heel” of CSCs for targeting and eliminating them. These approaches include chemotherapeutic drugs, TAA-specific antibodies, and gene and cell therapies. In accordance, Guerrero-Rodriguez et al. reviewed the potential value of the cell surface protein CD36 as a potential target for the development of novel strategies aimed at eradicating cancer stem cells and slowing tumor growth (Guerrero-Rodriguez et al.).

The regulation of the self-renewal of CSCs is orchestrated by specific signaling pathways. In this review article, the authors summarize the current knowledge on the main signal pathway governing breast cancer stem cell (BCSCs) self-renewal and the challenges associated with harnessing these signal pathways as therapeutics for the treatment of breast cancer. The authors discuss the key signaling pathways involved including the Wnt, NF-κB, Notch, Hedgehog, Hippo, and the TGF- *β* signaling pathways. It is important to note that the regulation of BCSCs is complex, and the interplay of multiple signaling pathways can vary among different breast cancer subtypes (Ordaz-Ramos et al.). Combination therapies targeting multiple pathways and personalized medicine approaches are being explored to address BCSCs and improve breast cancer treatment outcomes.

The canonical Wnt cell signaling is regulated by a plethora of molecules including SFPR1 (secreted frizzled-related protein 1). Losada-Garcia et al. conducted a study to demonstrate that SFPR1 regulates canonical Wnt signaling and favors a stem cell phenotype in prostate cancer. Their findings indicate that exogenous SFRP1 treatment expands the prostate cancer stem cell fraction and elevates the expression of cancer stem cell markers and genes related to the Wnt/β-catenin pathway. The authors demonstrate that SFRP1 regulates the subpopulation of CSCs by modulating the Wnt-beta catenin signaling pathway (Losada-Garcia et al.). This study stimulates further investigations aimed at discerning the potential of SFRP1 as a target for the elimination of prostate cancer stem cells.
